# Systematic Identification and Characterization of *O*-Methyltransferase Gene Family Members Involved in Flavonoid Biosynthesis in *Chrysanthemum indicum* L.

**DOI:** 10.3390/ijms251810037

**Published:** 2024-09-18

**Authors:** Man Zhang, Tao Wang, Qiaosheng Guo, Yong Su, Feng Yang

**Affiliations:** Institute of Chinese Medicinal Materials, Nanjing Agricultural University, Nanjing 210095, China; 2019204055@njau.edu.cn (M.Z.); wt1344@njau.edu.cn (T.W.); 2020204056@stu.njau.edu.cn (Y.S.); 2018204047@njau.edu.cn (F.Y.)

**Keywords:** Chrysanthemum, *O*-methyltransferase, flavonoids, *CiCCoAOMT1*

## Abstract

*Chrysanthemum indicum* L. capitulum is an enriched source of flavonoids with broad-ranging biological activities, mainly due to their anti-inflammatory, anti-cancer, immune regulation, anti-microbial activity, hepatoprotective, and neuroprotective effects. The *O*-methylation of various secondary metabolites has previously been demonstrated to be mainly catalyzed by *S*-adenosyl-L-methionine-dependent *O*-methyltransferase (OMT) proteins encoded by the *OMT* gene family. However, limited comprehensive study was published on the *OMT* gene family, especially the *CCoAOMT* subfamily, involved in the *O*-methylation of flavonoids in Chrysanthemum. Here, we analyzed the spatiotemporal expression patterns of *C*. *indicum OMT* genes in leaf and flower at different developmental stages. Transcriptome sequencing and qRT-PCR analysis showed that *COMT*s were mainly highly expressed in capitulum, especially in full bloom, while *CCoAOMT*s were mainly highly expressed in leaves. Correlation analysis of *OMT* gene expression and flavonoids accumulation revealed that four *OMT*s (*CHR00029120*, *CHR00029783*, *CHR00077404*, and *CHR00078333*) were putatively involved in most methylated flavonoids biosynthesis in the capitulum. Furthermore, we identified a true CCoAOMT enzyme, CiCCoAOMT1, and found that it catalyzed *O*-methylation of quercetin and luteolin at the 3′-OH position. In summary, this work provides an important theoretical basis for further research on the biological functions of *OMT*s in *C*. *indicum*.

## 1. Introduction

*Chrysanthemum indicum* L. is an economically important multi-purpose horticultural crop with medicinal and ornamental value. It is rich in flavonoids, and more than 60 methoxy flavonoids have been identified, among which chrysoeriol, acacetin, linarin, apigenin, and luteolin are the main anti-inflammatory components of *C*. *indicum* [1,2]. Flavonoids are widely distributed secondary metabolites in plants, with diverse biological activities, and are beneficial to human health. Plant flavonoids are important medicinal components [3], and their biological functions are closely related to chemical structures. *O*-methylation modification can increase the stability, protein affinity, and bioavailability of flavonoids, thereby enhancing their medicinal activities. Studies have confirmed that *O*-methylated flavonoids exhibit stronger antioxidant [4], anti-inflammatory [5], anti-cancer functions [6], higher bioavailability [7], and more medicinal value.

The *O*-methylation modification of plant flavonoids is catalyzed by *S*-adenosyl-L-methionine (SAM)-dependent *O*-methyltransferase gene (*OMT*) (EC 2.1.1). According to protein molecular weight and cation dependence, the plant OMT family can generally be classified as two distinct subfamilies, caffeoyl-CoA *O*-methyltransferase (CCoAOMT) and caffeic acid *O*-methyltransferase (COMT) [8]. The number of members of the COMT subfamily is relatively large, and the molecular weight of its protein subunits sizes is generally between 38 and 43 kDa, often forming homodimers, and the catalysis does not depend on cations [9,10]. The currently reported flavonoid OMT (FOMT) primarily belong to this subfamily [11]. The CCoAOMT subfamily has relatively few members, and its protein subunits sizes are relatively lower (23–30 kDa) and are mostly cation-dependent. Some of CCoAOMTs specifically catalyze caffeoyl-CoA, a key enzyme in the lignin biosynthesis pathway [12,13]. Based on substrate preference, the CCoAOMT subfamily is classified into two subgroups: phenylpropanoid and flavonoid *O*-methyltransferase (PFOMT) and true CCoAOMT. PFOMT, also known as CCoAOMT-like, has pronounced substrate preferences for caffeoyl-CoA and flavonoids, with special reference to anthocyanins and flavonols [14,15]. Although the true CCoAOMT subgroup is responsible for lignin biosynthesis, several members have been reported to be able to methylate flavonoids, such as IiOMT1 from *Isatis indigotica* [16], MpalOMT3 from *Marchantia paleacea* [17], and OsOMT26 from *Oryza sativa* [18].

*OMT* gene family member identification and functional characterization have been performed in multiple plant species such as *Arabidopsis thaliana* [19], *Citrus* [20], *Glycine max* (L.) Merr. [21], *Plagiochasma appendiculatum* [22], *Ocimum basilicum* [23], *Vitis vinifera* [24], and *Arachis hypogaea* [25]. The OMT proteins of different species have high homology, especially those with the same catalytic site and from the same family or genus. Consequently, using phylogenetic trees and functional identification, potential genes for flavonoid *O*-methylation can be predicted in silico [26]. Up to now, several *OMT* genes have been proved to be related to the methylation of flavonoids in the family Aizoaceae. For example, *CtMROMT* purified from safflower seeds has been demonstrated to methylate apigenin efficiently into acacetin [27]. Similar findings were found in other plants. *Pa4′OMT* (*Plagiochasma appendiculatum*) is a multifunctional *OMT* that contributes to converting flavonoids such as luteolin, naringenin, kaempferol, quercetin, genistein, scutellarein, and genkwanin to the corresponding methylation products [28]. *CmCCoAOMT1* (caffeoyl-CoA *O*-methyltransferase 1) in *Chrysanthemum morifolium* and *CgCOMT* (caffeic acid 3-*O*-methyltransferase) in *Chrysanthemum grandiflorum* (Ramat.) Kitamura may be related to lignin synthesis [29,30]. The methoxylated flavonoid chrysoeriol serves as a quality evaluation indicator for *C*. *indicum*. Some progress has been made in the study of *CiFNSII* [31] and *RhaT* [32] involved in flavone biosynthesis. However, few studies have performed a comprehensive investigation of CCoAOMTs involved in the biosynthesis of methoxylated flavonoid in *C*. *indicum*.

In this study, we investigated the *OMT* gene family at the genome-wide level and potential members involved in flavonoids methylation in *C*. *indicum* based on published Chrysanthemum genome sequences. Moreover, we analyzed transcriptome data on *C*. *indicum* and identified a true CCoAOMT enzyme, CiCCoAOMT1, catalyzing *O*-methylation of flavonoids at the 3′-OH position in vitro and in vivo assays. The research results conduce to understand the characteristics of the *OMT* gene family in *C*. *indicum*, in particular, and provide useful information for screening the members involved in *O*-methylated flavonoids synthesis.

## 2. Results

### 2.1. Identification and Sequence Analysis of OMT Genes in C. indicum

In the Chrysanthemum genome, 31 and 48 genes encoding CCoAOMTs and COMTs, respectively, were identified and named as *CiCOMT*s, *CiCCoAOMT*s. All sequence characteristics were evaluated and the details are summarized in Table S1. The amino acids number of the COMT proteins ranged from 192 to 447, while those of CCoAOMT proteins ranged from 175 to 305. The MW of COMT proteins varied between 21.14 and 50.00 kDa and the pI varied between 4.67 and 8.25, whereas for CCoAOMT proteins, the MW varied from 20.24 to 35.43 kDa, and the pI varied from 4.50 to 9.88. The instability index of COMTs varied from 22.31 to 44.77. The instability indices of COMTs and CCoAOMTs were similar, ranging from 22.31 to 44.77 and 24.98 to 44.28, respectively. The number of negative GRAVY for COMT and CCoAOMT proteins is 32 (67%) and 33 (97%), respectively, indicating that they are mostly hydrophilic proteins. *COMT* and *CCoAOMT* genes tended to consist of two exons, with methylation domains about 210 bp in length. According to the predicted subcellular localization results, OMTs were mostly localized in the cytoplasm, while a few were located in the chloroplast, cytoskeleton, endoplasmic reticulum, or nucleus.

### 2.2. Phylogenetic Analysis of OMTs

**Figure 1:**
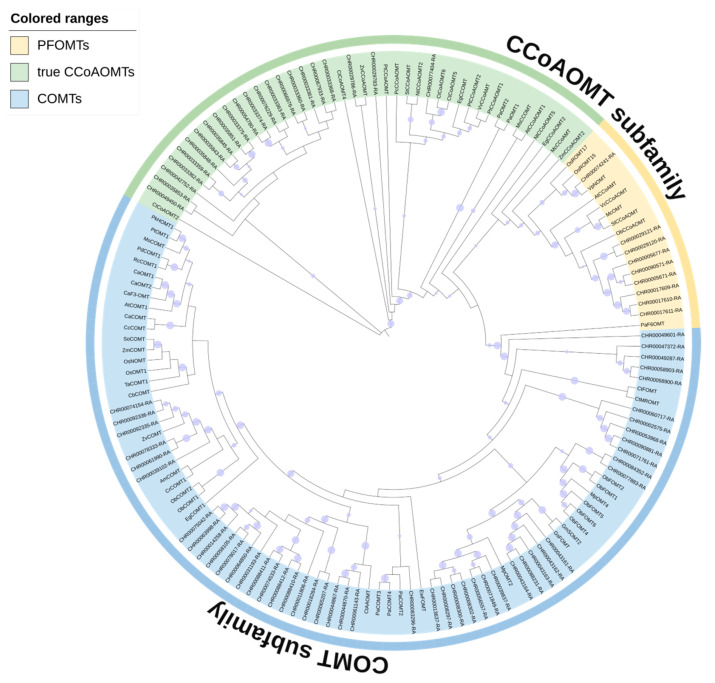
Phylogenetic relationships of OMTs in different species. Subclades are marked by different backgrounds. The blue arc represents COMT subfamily; green and yellow arcs represent CCoAOMT subfamily. UniProt entries for these OMTs are given in Table S2.

An unrooted phylogenetic tree (Figure 1) was constructed using the full-length protein sequences found in *C*. *indicum*, *Arabidopsis thaliana*, *Oryza sativa*, *Ocimum basilicum*, etc., to explore the evolution of the OMT family. The corresponding bootstrap values are represented by a purple circle on the clade (bootstrap value > 50 is displayed, and the size of the circle indicates the high and low values).

The phylogenetic tree clearly shows that OMT proteins are divided into two subfamilies based on their sequence similarity and topology, including the COMT subfamily (blue shading) and the CCoAOMT subfamily, of which 79 *C*. *indicum* OMTs are distributed in the two subfamilies. The CCoAOMT subfamily of the reference species can be further divided into the PFOMT subgroup (yellow shading) and the true CCoAOMT subgroup (green shading). Inferred from the homology between species, the *C*. *indicum* CCoAOMTs in yellow and green shadows may have correspondingly similar biological activities. The *C*. *indicum* OMTs were spaced in distribution from other plant OMTs on the phylogenetic tree, indicating that the OMT family is highly conserved during plant evolution.

### 2.3. Gene Structure and Protein Conserved Motifs of C. indicum OMT Genes Family

**Figure 2:**
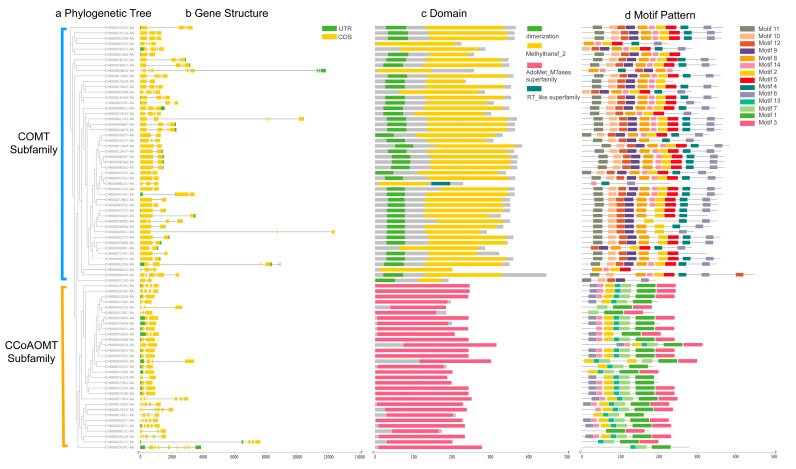
Phylogenetic relationship, gene structure, and motif analysis of *C*. *indicum OMT*s. (**a**) Phylogenetic tree. (**b**) Gene structure analysis. Exons (CDS, coding sequence) and introns are represented by the yellow box and the grey line, respectively. The green box represents UTR (untranslated region). (**c**) Conserved domain prediction. The protein length can be estimated using the scale at the bottom. (**d**) Conserved motif prediction. Motifs 1–14 are indicated by the different color boxes.

The phylogenetic tree of *C*. *indicum OMT*s was interactively analyzed with its gene structure and protein structure, and the differences in gene structure and protein structure were explored from an evolutionary perspective. Consistent with the results of the evolutionary relationship analysis in Section 2.2, the OMT family can be clearly divided into two clades: the COMT subfamily and the CCoAOMT subfamily (Figure 2a). The Chrysanthemum genome was assembled to the scaffold level, with a contig N50 of 130.7 kb, which is much larger than 10 kb, indicating good contiguity [33]. Gene structure analysis revealed that the exons and introns count in *OMT* genes are varied (Figure 2b). Exon numbers of *CiCCoAOMT*s ranged from 4 to 10, while those of *CiCOMT*s ranged from 2 to 9. Both of them typically contained two exons and one intron. Due to the limitation of genome assembly to the scaffold level, chromosomal locations analysis cannot be performed, and intron–exon patterns reflect the *OMT*s gene structure to some extent. Despite exon sizes being fairly conserved, the length of untranslated regions varied greatly.

Conserved domain analysis showed that the amino acid sequences of CiCOMT proteins were relatively long, and they all contained the conserved domain Methyltransf_2 (Pfam: pfam00891) of *O*-methyltransferase. The *N*-terminus of most CiCOMT members contained a dimerization domain (Pfam: pfam 08100), which plays an important role in the formation of protein dimers. In addition, COMT member CHR00088231 also contains an RT_like superfamily domain. CiCCoAOMT proteins have relatively short amino acid sequences and contains only one domain, the AdoMet_Mtases superfamily, which is a conserved domain of class I methyltransferases (Figure 2c).

Fourteen conserved motifs were identified and labeled as motifs 1–14 (Figure 2d, Table 1). Based on MEME results, the majority of CiCOMT proteins contained ten conserved motifs. However, some proteins such as CHR00088231 only had motifs 4, 6, and 14. The number of motifs found in CiCCoAOMT was relatively small, among which motif 7 is completely conserved. CiCCoAOMT proteins were widely distributed with motifs 1, 3, and 13, indicating that these motifs may be important for the function of the protein.

According to these findings, *OMT*s have different structural patterns, and members of the same group have similar exon–intron architecture and motifs composition.

### 2.4. Expression Patterns of C. indicum OMTs Based on the RNA-seq Data

**Figure 3:**
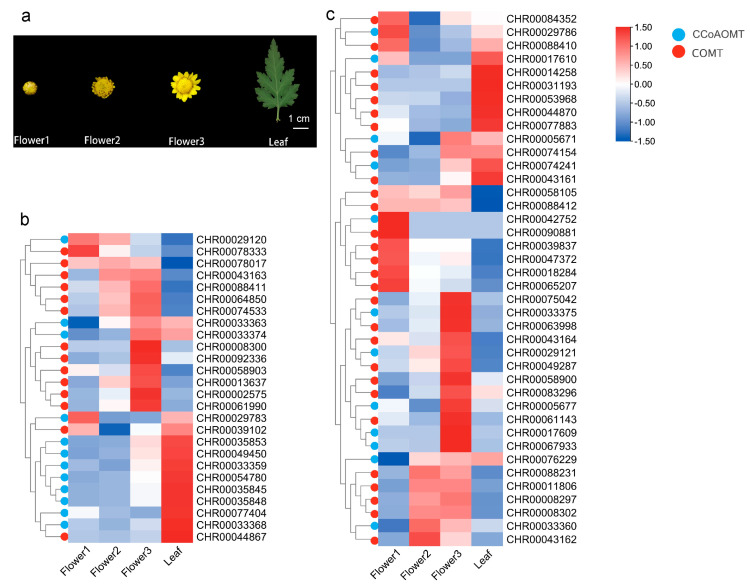
Expression pattern of *OMT*s in different capitulum development stages and leaves of *C*. *indicum*. (**a**) *C*. *indicum* tissues. Flower1–3, respectively, correspond to flower bud stage, early flowering stage, full opening stage. Heatmap display of *C*. *indicum OM*Ts with relatively high transcript levels (FPKM > 10) (**b**) and with relatively low transcript levels (FPKM < 10) (**c**). The original data of the RNA-seq are shown in Table S3, and the hierarchical clustering analysis (Figure S1) shows its reproducibility and reliability. The color scale from blue to red color represents Z-score-normalized gene expression levels from low to high. Dendrograms on the left side of the heat map show the hierarchical clustering between genes. At the tip of the cluster tree, *CiCOMT*s and *CiCCoAOMT*s are marked with red and blue circle dots, respectively.

Looking into the expression profiles of *C*. *indicum OMT* genes is essential to determine how they affect the synthesis of flavonoids. The RNA-Seq data of the capitulum and leaves of *C*. *indicum* (Figure 3a) were used to explore spatiotemporal expression patterns of *OMT*s. The heatmap hierarchical clustering analysis (Figure 3b) revealed that across the observed samples, the expression profiles of *OMT* members differed greatly. Among them, twenty-six *OMT* genes (33% of all *C*. *indicum OMT* genes) were expressed in relatively high abundance (FPKM > 10 for at least one stage), including fourteen *COMT*s and twelve *CCoAOMT*s. Only nine *OMT* genes with the highest expression abundance (FPKM > 50 for at least one stage) were *CHR00013637*, *CHR00029783*, *CHR00033359*, *CHR00039102*, *CHR00043163*, *CHR00064850*, *CHR00074533*, *CHR00077404*, *CHR00088411*. Most *COMT*s showed relatively high expression in the capitulum, with the highest transcript levels at the flower3 stage, and were clustered together in the heatmap (Figure 3b). *CHR00013637*, *CHR00002575*, *CHR00043163*, *CHR00061990*, *CHR00074533*, and *CHR00078333* only showed expression in the capitulum. Most *CCoAOMT*s showed high expression in leaves, such as *CHR00049450*, *CHR00035853*, *CHR00033359*, *CHR00054780*, *CHR00035845*, *CHR00035848*, *CHR00077404*, and *CHR00033368*. There were forty *OMT* genes (51% of all *C*. *indicum OMT* genes) with relatively low expression abundance (FPKM value < 10 at each stage), of which twenty-eight were *COMT* members and twelve were *CCoAOMT* members (Figure 3c). Furthermore, no transcript of thirteen *OMT* genes (16% of all *C*. *in dicum OMT* genes) was detected.

### 2.5. qRT-PCR Analysis of C. indicum OMTs Expression in Various Tissues

**Figure 4:**
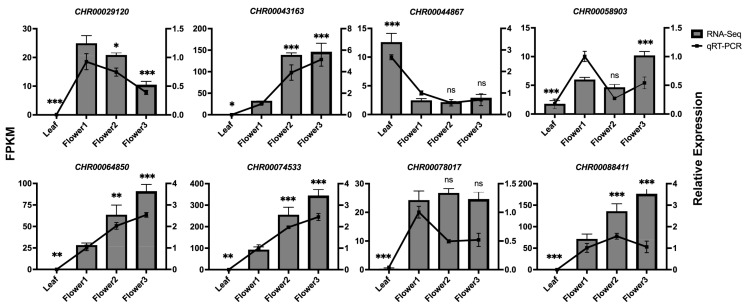
Quantitative expression and relative expression levels of *OMT*s in capitulum at different development stages and leaves of *C*. *indicum*. The gene name appears at the top of each histogram and tissues appear at the bottom. The relative expression level of each gene in flower1 is set to 1. Error bar means standard deviation (SD) among three independent replicates. * *p* < 0.05, ** *p* < 0.01, *** *p* < 0.001, ns means no significance.

To validate the RNA-seq data, we performed qRT-PCR assays with independent samples collected from the leaves and capitulum at different developmental stages. It has been determined that a small subset of plant *CCoAOMT*s known as *PFOMT*s exhibit substrate selectivity for phenylpropanoids such as flavonoids [8]. The percent identity between *CHR00029120* (*CCoAOMT*) and *ObCCoAOMT* was 61.37%, higher than 50%, as performing Align Sequences Protein BLAST in NCBI. Meanwhile, according to the phylogenetic tree (Figure 1), it is speculated that *CHR00029120* may be homologous to *ObCCoAOMT*, which has been characterized by exhibiting a substrate preference for flavonoids. *CHR00029120* (*CCoAOMT*) and a few *COMT* genes with relatively high transcript levels (FPKM > 50) were chosen based on the study above for qRT-PCR expression detection in different tissues and growing capitulum. The primers for these selected genes are listed in Table S4 and the melting curve shows a single peak (Figure S2), confirming the amplification specificity. The qRT-PCR analysis results are presented in Figure 4. Six *OMT* genes (*CHR00029120*, *CHR00043163*, *CHR00064850*, *CHR00074533*, *CHR00078017*, and *CHR00088411*) showed a nearly non-detectable transcript level in leaves, i.e., specifically expressed in the capitulum. These genes’ expression patterns could be roughly divided into three categories. Pattern one comprised one gene (*CHR00029120*) that was downregulated with the capitulum developmental process, reaching a low level at the flower3 stage. Pattern two included four genes (*CHR00043163*, *CHR00064850*, *CHR00074533*, and *CHR00088411*) that were upregulated during capitulum development and had high transcript levels at the flower3 stage, which was opposite to the pattern one expression trend. Pattern three included one gene (*CHR00078017*) with a relatively stable transcript concentration throughout capitulum development. In all tissues detected, *CHR00044867* and *CHR00058903* were constitutively expressed. The former was strikingly upregulated in the leaves, while the latter showed fluctuating expression. The qRT-PCR results for these chosen genes were essentially similar to the RNA-seq results in terms of their expression levels.

### 2.6. Identification of OMT Genes Involved in Flavonoids Synthesis during the Development of C. indicum capitulum

**Figure 5:**
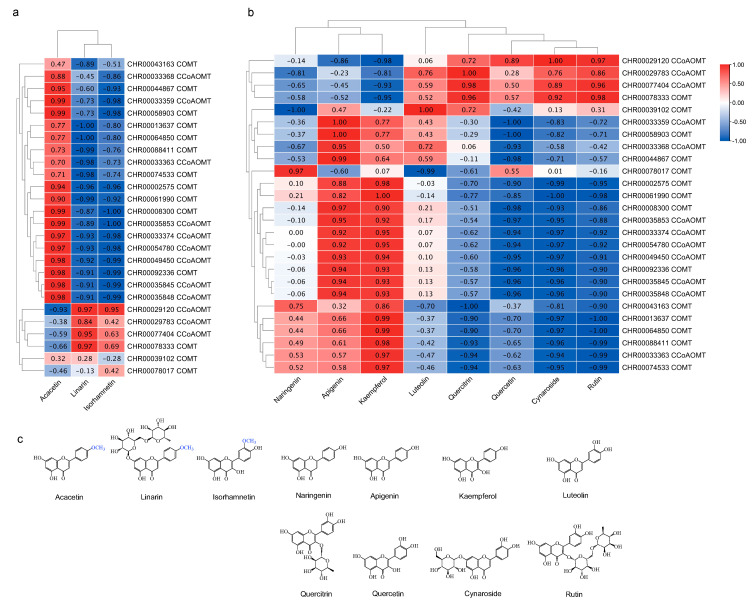
Screening of *C*. *indicum OMT* genes involved in flavonoid accumulation in the capitulum. (**a**) Correlation analysis heatmap between methylated flavonoid concentrations content and the expression of *C*. *indicum OMT* genes (FPKM > 10 in at least one sample) based on Pearson correlation coefficient (r). (**b**) Correlation analysis heatmap between potential methylation substrates content and the expression of *OMT* genes based on Pearson correlation coefficient (r). (**c**) Flavonoid structure. Methylation sites are marked in blue.

To further screen potential *OMT* genes involved in methylated flavonoid accumulation, we analyzed the Pearson correlation coefficient (r) between methylated flavonoids content and the relative expressions of *OMT*s, and visualized the data with TBtools; the result is presented in Figure 5a,b. Three methylated flavonoids with anti-inflammatory activity, namely acacetin, linarin, and isorhamnetin, and eight potential methylation substrates, including cynaroside, quercitrin, luteolin, quercetin, apigenin, naringenin, and kaempferol in *C*. *indicum* capitulum were tested using UPLC, and rutin was determined by UV spectrophotometry (Figure 5c). The results are presented in Table S5. There were significant differences in its flavonoids content. The highest content was found in the flower1 stage, and the lowest was found in the flower3 stage. Some flavones and flavonols also exhibit similar changes, that is, they were enriched and accumulated in the flower1 stage, while luteolin and quercetin contents were almost similar in each stage. Of the tissues examined, only 26 of 79 *OMT*s, including 12 *CCoAOMT*s and 14 *COMT*s, were expressed at relatively high levels (FPKM >10 in at least one sample). An expression-concentration correlation coefficient of more than 0.7 indicated that the gene may be involved in methylated flavonoid synthesis in the capitulum. According to the results, *CHR00029120*, *CHR00029783*, *CHR00077404*, and *CHR00078333* expression levels were highly correlated with linarin, isorhamnetin, and some potential methylation substrates, such as quercitrin, quercetin, rutin, and cynaroside, which were highly accumulated at the flower1 stage. The expression level of *CHR00078017* correlated strongly with the accumulation of naringenin, which was highly accumulated at the flower2 stage. The highly expressed *OMT* genes *CHR00043163*, *CHR00013637*, *CHR00064850*, *CHR00088411*, and *CHR00074533* correlated strongly with the accumulation of kaempferol and apigenin. Moreover, *CHR00033359*, like the rest of the *OMT*s, correlated strongly with the accumulation of kaempferol, apigenin, and acacetin, which were highly accumulated at the flower3 stage. *CHR00039102* showed a high correlation with luteolin (Figure 5b), and there was no significant difference in the accumulation of luteolin at different capitulum development stages. These data implied that *CHR00029120*, *CH00029783*, *CHR00077404*, and *CHR00078333* may play a key role in the synthesis of most flavonoids in *C*. *indicum* capitulum, especially methylated flavonoids.

### 2.7. Heterogeneous Expression of CiCCoAOMT1 in E. coli and In Vitro Enzymatic Activity Assays

**Figure 6:**
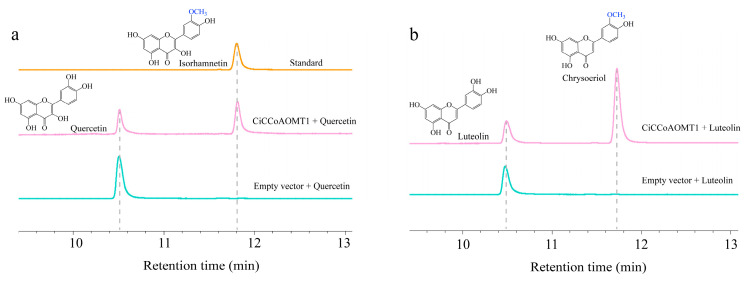
UPLC chromatograms of the reactions of CiCCoAOMT1 with different flavones substrates including quercetin (**a**) and luteolin (**b**). Substrates incubated with the empty vector (pET32a) are indicated in cyan. Substrates utilized by recombinant CiCCoAOMT1 are indicated in red. Authentic compounds of methylated products are indicated in yellow. OCH3 in blue color represents the methylation site. The interrupted line points to the retention time of the compound.

The open reading frame (ORF) of *CiCCoAOMT1* gene is 744 bp encoding 247 amino acids, and the molecular weight (MW) of predicted protein is 27.8 kDa. The CiCCoAOMT1 protein with a hexahistidine (His)-tag, an S-tag, and a Trx-tag at the *N*-terminus was highly expressed in *E*. *coli* BL21 (DE3). The purified proteins were analyzed by SDS-PAGE and formed a band of about 47 kDa on the gel, which was consistent with the theoretical MW of 50.3 kDa (Figure S3). CiCCoAOMT1 activity was characterized *in vitro* with a wide range of potential substrates (Figure 5c) in the presence of SAM. As shown in Figure 6, CiCCoAOMT1 efficiently converted quercetin into a single methylated product, isorhamnetin (Figure 6a), and luteolin was presumed to be converted to chrysoeriol (Figure 6b) [15,16]. No methylation product was observed when several other phenolic compounds were used as substrates. The results suggested that the CiCCoAOMT1 could be involved in flavonoid biosynthesis.

### 2.8. Subcellular Localization Analysis of CiCCoAOMT1 Protein

**Figure 7:**
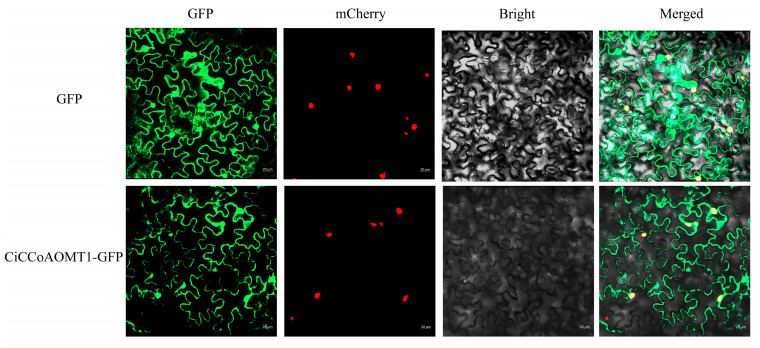
Subcellular localization of CiCCoAOMT1 in *N*. *benthamiana*. GFP, GFP channel; mCherry, RFP channel; Bright, blight field channel; Merged, merged image of the GFP, mCherry, and Bright channels. Scale bars are 20 μm.

To examine the localization of CiCCoAOMT1 in plant cells, recombinant CiCCoAOMT1 with the GFP tag was overexpressed in *N*. *benthamiana*. As can be seen from Figure 7, the fluorescence signal distribution pattern of CiCCoAOMT1-GFP fusion protein was similar to that of the empty vector, mainly located in the membrane and nucleus, indicating that CiCCoAOMT1 is a membrane and nuclear localization protein.

### 2.9. Overexpression of CiCCoAOMT1 in C. indicum

**Figure 8:**
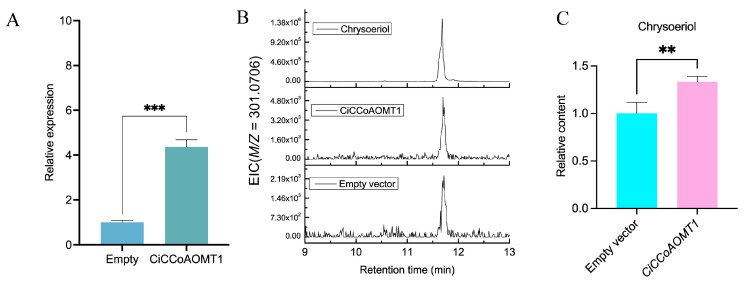
Overexpression analysis of *CiCCoAOMT1* in *C*. *indicum*. (**A**) The expression pattern of *CiCCoAOMT1*. (**B**) LC-MS extracted ion chromatograms (EIC) of *C*. *indicum* with CiCCoAOMT1 or empty vector. (**C**) Changes in the chrysoeriol content after overexpression of *CiCCoAOMT1* gene. Data are mean ± standard deviation of three biological replicates. ** *p* < 0.01, *** *p* < 0.001 (1-tailed paired *t*-test).

In order to further study the catalytic activity of CiCCoAOMT1, the leaves of *C*. *indicum* tissue culture seedlings were infected with *Agrobacterium* suspension containing *CiCCoAOMT1*-pCAMBIA1300-cGFP and empty vector (pCAMBIA1300-cGFP), respectively. Transgenic calli were obtained through genetic transformation processes such as co-culture, decarboxylation, and selective culture. The potential role of CiCCoAOMT1 in flavonoid methylation in vivo was revealed by qRT-PCR and LC-MS metabolite analysis. The results showed that the transcription level of *CiCCoAOMT1* was significantly upregulated by 4.4 times compared with the control group (Figure 8A). As expected, the expression of *CiCCoAOMT1* in *C*. *indicum* resulted in a significant accumulation of methoxyflavone. Compared with the control group injected with empty vector, the content of chrysoeriol significantly increased by 1.33-fold (Figure 8C). Taken together, CiCCoAOMT1 is a potential candidate for chrysoeriol production through 3ʹ-*O*-methylation of piceatannol in the B-ring. However, further *CiCCoAOMT1* overexpression transgenic lines in vivo were still needed to investigate its role in the biosynthesis of methylated flavonoids in planta.

## 3. Discussion

OMTs play a significant role in plant secondary metabolism, such as methylating the oxygen atom of flavonoids [34]. The characteristics and functions of some *OMT* genes have been studied in plants such as *Arabidopsis* [35,36], soybean [37,38], and wheat [39]. *C*. *indicum* is a natural resource rich in flavonoids, which are intimately associated with the medicinal and nutritional value of the capitulum [40]. Taking into account the significance of flavonoids in *C*. *indicum* and the lack of information regarding their methylations, this project utilized the Chrysanthemum genome and RNA-seq data to characterize *OMT* genes related to flavonoid biosynthesis.

### 3.1. Interspecific Divergence at the Scale of OMTs

There are large differences in the number of *OMT* members in various species. In this paper, a total of 79 putative *OMT* members were identified in Chrysanthemum, significantly greater than those in *Citrus sinensis* (58) [20], *V*. *vinifera* (47), *A*. *thaliana* (24), and *P*. *trichocarpa* (26) [41] according to incomplete statistics, suggesting Chrysanthemum OMTs have undergone expansion. The expansion of the *OMT* gene family may diversify the mechanisms required for plant adaptation to the environment. Previous studies have shown that *COMT*s likely assisted in plant terrestrialization and their adaption to terrestrial ecosystems [42]. Whole-genome duplication (WGD) is regarded as the primary driving force for gene family expansion and evolution, validated in model plants *Arabidopsis* and *Populus* [43,44,45]. Additionally, the kiwifruit *COMT*s have undergone two WGD events [46]. Similarly, the PgAOMT family has evolved and expanded primarily through WGD and tandem duplication, resulting in new or non-functionalized *PgAOMT*s [47]. Research shows that the most recent WGD event occurred in the Chrysanthemum genome approximately 38.8 million years ago [33].

### 3.2. Phylogenetic Relationship and Classification of OMTs

Phylogenetic analysis of plant OMT protein sequences from different species showed that they were distributed in two major lineages based on functional traits reflecting their substrate specificity, one including CCoAOMT proteins using cateyl-CoA derivatives as substrates, and the other including COMT protein sequences using simple phenols, flavonoids, and alkaloids as substrates [48]. The phylogenetic distribution in this study showed that 135 OMT proteins from Chrysanthemum and other plants were clustered into two groups (Figure 1). Consistent with the characteristic features of the *OMT* gene family, the *COMT* subfamily contained more members than the *CCoAOMT* subfamily, which may explain their multiple functions and participation in a diverse set of physiological functions in plant development. In addition, the distribution characteristics of OMT-binding motifs support this classification with COMT proteins having more motif types (Figure 2d). The results of phylogenetic tree analysis and protein structure analysis of Chrysanthemum *OMT* genes showed that genes with similar gene structures and protein structures were more closely related in the phylogenetic tree (Figure 2a), which may be closely related to the tandem duplication and chromosome segment duplication during the expansion of Chrysanthemum *OMT* gene family association [20]. However, some closely related *OMT* members have large differences in their gene expression patterns. The expression of 11 Chrysanthemum *OMT* genes was not detected in all the tested tissues, and this phenomenon also existed in some citrus *OMT* genes [20]. It is speculated that these genes may be loss-of-function or only expressed under certain conditions or at certain developmental stages.

### 3.3. Tissue-Specificity in OMTs and Flavonoids Accumulation

Differences in the expression patterns of *OMT* gene family members are generally considered to be related to their specific biological functions. *FOMT*s identified in sweet basil [23] and tomato [49] typically exhibit tissue-specific, primarily expressed in leaf glandular hairs enriched with methylated flavonoids. While the major role of the *CCoAOMT* family was originally attributed to lignin biosynthesis, some plant *CCoAOMT* genes such as *VvCCoAOMT4* [24], *PaF6OMT* [50], *ObCCoAOMT* [51], and *McOMT* [14] encode proteins that exhibit a substrate preference for flavonoids. Proteins that are more closely clustered are more likely to have similar functions. Considering the possibility of homology between *CHR00029120* (*CCoAOMT*) and *ObCCoAOMT* (Figure 1), it is speculated that the two perform similar or related functions. Furthermore, *CHR00029120* expression level was indeed highly associated with methoxylated flavonoid linarin and isorhamnetin (Figure 5a). According to the expression heatmap (Figure 3b), *CHR00029120* and *CHR00029783* were consistently downregulated during capitulum development, as confirmed by qRT-PCR (Figure 4), suggesting that they primarily function at the flower bud stage. It is intriguing that the *CHR00029120* transcripts were barely detectable in leaves and were most abundant in the capitulum, demonstrating that some specific *O*-methylated flavonoids or other secondary metabolites might be formed in the capitulum [20]. However, the specific function still needs further experimental analysis to confirm. In contrast, both *CHR00077404* and *CHR00077883* showed higher expression levels in leaves. Additionally, only nine *OMT* genes (three *CCoAOMT*s, six *COMT*s) exhibited abundant expression (FPKM > 50) in at least one tissue or developmental stage (Figure 3b). Except for *CHR00029783* (*CCoAOMT*) and *CHR00077404* (*CCoAOMT*), most other genes were only highly correlated with the methoxylated flavonoid acacetin in the correlation analysis (Figure 5a). The differential expression of these *OMT* genes is particularly important for for us to understand the mechanisms of flavonoids metabolism during capitulum development.

### 3.4. CiCCoAOMT1 Is Involved in the Methylation of Flavonoids

In the present study, we identified a member of the true CCoAOMT subgroup from *C*. *indicum* (CHR00029783) and named it CiCCoAOMT1. CiCCoAOMT1 recombinase showed high affinity and catalytic efficiency for flavonoid substrates in vitro, converting 3′-OH of flavonoids with vicinal hydroxyl groups to the corresponding monomethyl ethers and validated in vivo. Similarly, this catalytic activity has been verified in vivo in several OMTs such as IiOMT1, IiOMT2 and CsCCoAOMT1 from *Isatis indigotica* [16] and *C*. *sinensis* [15], respectively. Combined with the result of protein subcellular localization in *N*. *benthamiana* leaf epidermal cells, CiCCoAOMT1 might be involved in the methylation of flavonoids in the cytoplasm of the plant cell. A similar localization pattern was also observed in the true CCoAOMT subgroup, such as VvCCoAOMT4 from *V*. *vinifera* [24], and CsCCoAOMT1 from *C*. *sinensis* [15].

## 4. Materials and Methods

### 4.1. Plant Materials

The capitulum and leaves of *C*. *indicum* were used in this study, which were obtained from the *C*. *indicum* germplasm resource nursery of Nanjing Agricultural University. There were flower1 (at flower bud stage), flower2 (at the early flowering stage), flower3 (at the full opening stage), leaf (leaves), harvested in November 2021. After freezing in liquid nitrogen, the materials were stored at −80 °C for RNA isolation and flavonoid analysis.

### 4.2. Acquisition and Analysis of Transcriptome Sequencing

Total RNA was extracted using Plant RNA Purification Reagent (TaKaRa, Beijing, China), and RNA quality was assessed on a Nano 300 micro-spectrophotometer (Allsheng, Hangzhou, China) and 1% agarose gel. Raw reads were obtained by sequencing on the DNBSEQ platform by BGI Shenzhen Co., Ltd., and processed using SOAPnuke (v1.4.0) (https://github.com/BGI-flexlab/SOAPnuke accessed on 20 February 2022) to obtain high-quality clean reads, which were then compared to the Chrysanthemum genome (http://210.22.121.250:8880/asteraceae/homePage accessed on 20 February 2022) using HISAT (v2.1.0) (http://www.ccb.jhu.edu/software/hisat accessed on 20 February 2022). Subsequently, novel transcripts were reconstructed and integrated using StringTie (v1.0.4) (http://ccb.jhu.edu/software/stringtie accessed on 20 February 2022) and Cuffmerge (v2.2.1) (http://cole-trapnell-lab.github.io/cufflinks accessed on 20 February 2022). Protein coding potential prediction was performed using CPC (v0.9-r2) (http://cpc.cbi.pku.edu.cn accessed on 20 February 2022). Finally, the novel transcripts with protein coding potential were added to the reference gene sequence, forming a complete reference sequence. Gene expression levels were calculated using RSEM (v1.2.8) (http://deweylab.biostat.wisc.edu/rsem/rsem-calculate-expression.html accessed on 20 February 2022) based on FPKM (fragments per kilobase of exon model per million mapped fragments).

### 4.3. Data Sources and Gene Identification of OMTs

Whole-genome proteins sequence files of *C*. *indicum* were downloaded from the Chrysanthemum Genomic Database (http://210.22.121.250:8880/asteraceae/homePage accessed on 7 June 2022). Conserved methyltransferase domains were downloaded from Pfam (http://pfam.xfam.org accessed on 7 June 2022) by retrieving PF01596 (CCoAOMT) and PF00891 (COMT) and used as Hidden Markov Model (HMM) queries to search potential *C*. *indicum* OMT proteins with an e-value cutoff of 10^−6^ [52]. The integrity of the sequence feature domains was verified by NCBI Batch CD-search (https://www.ncbi.nlm.nih.gov/Structure/bwrpsb/bwrpsb.cgi accessed on 17 June 2022) and the SMART (http://smart.embl-heidelberg.de accessed on 17 June 2022) online database, and sequences without the target domains were removed. The physical and chemical properties of all *C*. *indicum OMT* gene families were available online on ExPASY (https://web.expasy.org/protparam/ accessed on 17 June 2022) [53], including molecular weight (MW), isoelectric point (PI), instability index, and the grand average of hydropathicity (GRAVY) values. The prediction of subcellular localization of the identified OMT proteins was performed with WoLF PSORT [54].

### 4.4. Sequence Alignment and Phylogenetic Analysis

The phylogenetic relationships of the identified 79 Chrysanthemum OMTs with 56 OMTs from other species such as mouse-ear cress, safflower, rice, and sweet basil was explored. These identified OMT protein sequences were utilized to perform multiple sequence alignment analysis with MAFFT version 7 (https://mafft.cbrc.jp/alignment/server/index.html accessed on 17 June 2022). The unrooted phylogenetic tree was constructed using the neighbor-joining (NJ) algorithm based on 1000 bootstrap replicates, visualized and annotated at iTOL (https://itol.embl.de/ accessed on 20 June 2022). The OMT protein sequences of other species were downloaded from the Unified Protein Database (https://www.uniprot.org accessed on 20 June 2022), and the UniProt entries (Accessed on 20 June 2022) for these OMTs are given in Table S2.

### 4.5. Exon–Intron Structure and Conserved Motif Analysis

The *C*. *indicum* OMTs phylogenetic tree was constructed according to the method in 4.4. The GFF3 format file containing DNA and CDS position information was downloaded from the Chrysanthemum genome database (https://cbcb.cdutcm.edu.cn/AGD/genome/details/?id=0005 accessed on 7 June 2022) to analyze the exon-intron structure. To further identify and analyze the protein conserved motifs of the *C*. *indicum* OMT family members, the Multiple EM for Motif Elicitation (MEME) (https://meme-suite.org/meme/tools/meme accessed on 20 June 2022) [55] was employed with the parameters that the maximum motif number was 14, and other parameters are default settings. Finally, TBtools (v 1.09876) was used to interactively visualize the above analysis results.

### 4.6. Expression Analyses of C. indicum OMT Gene Family Members

The abundances of transcripts were assessed by fragments per kilobase of transcript per million (FPKM) value mapped reads to quantify gene expression. FPKM values were visualized as heat maps using TBtools (version 1.098). FPKM values for *OMT* genes are provided in Table S3.

### 4.7. Total RNA Extraction and qRT-PCR Analysis

The total RNA extraction process and extraction stage are consistent with those in Section 2.2. Primer 5.0 was used to design primers (Table S4), and cDNA was synthesized from RNA using the TSINGKE TSK301S Goldenstar™ RT6 cDNA Synthesis Kit (Tsingke Biotechnology Co., Ltd., Beijing, China). *GAPDH* (registration number KC508619) and *EF1α* (registration number KF305681) were selected as the internal reference gene [56,57]. qRT-PCR analysis was conducted on a StepOnePlus™ Real-Time PCR System (Thermo Fisher Scientific, MA, USA) according to the instructions of the PrimeScript™ RT reagent Kit with gDNA Eraser (Takara Biomedical Technology (Beijing) Co., Ltd., Beijing, China). The correlative expression data were calculated according to the 2^−(∆∆CT)^ method with three biological repeats of each sample.

### 4.8. Correlation Analysis

Ultra-performance liquid chromatography (UPLC) was used for the content determination of 11 flavonoids. The samples were dried at 60 °C and ground into powder. Powder samples (0.5 g) were immersed in methanol (50 mL) for 30 min and then processed by ultrasonic extraction for 30 min. The extract was filtered by a 0.22 µm microporous nylon filter. Then, 2 µL of the filtrate was run on a Acquity UPLC (Waters Corporation, Milford, MA, USA) system with an ACQUITY UPLC HSS T3 column (2.1 × 100 mm, 1.8 µm, Waters Corporation, Milford, MA, USA). Acetonitrile (solvent A) and 0.1% phosphate solution (solvent B) comprised the mobile phase, and the gradient elution procedure was set up as follows: 0 min, 0% A/100% B; 3 min, 0% A/100% B; 5 min, 25% A/75% B, 8 min, 45% A/55% B; 15.5 min, 27% A/73% B; and 18.5 min, 0% A/100% B. The detection wavelength was set at 350 nm, and the flow rate was performed at 0.2 mL/min. The standards were purchased from Shanghai Yuanye Bio-Technology Co., Ltd. (Shanghai, China), including chlorogenic acid (B20782, 327-97-9), isochlorogenic acid A (B21539, 2450-53-5), caffeic acid (B20660, 331-39-5), apigenin (B20981, 520-36-5), luteolin (B20888, 491-70-3), acacetin (B20627, 480-44-4), linarin (B20860, 480-36-4), cynaroside (B20887, 5373-11-5), quercetin (B20527, 117-39-5), quercitrin (B20526, 522-12-3), kaempferol (B21126, 520-18-3), isorhamnetin (B21554, 480-19-3), and naringenin (B21596, 480-41-1). Statistical analysis was conducted using Microsoft Excel (version 2019). We explored the correlation between *OMT* gene expression and flavonoid concentrations using Pearson correlation coefficient analysis. The results were visualized with TBtools (version 1.098).

### 4.9. Heterologous Expression, and Purification of Recombinant CiCCoAOMT1 Proteins

The full coding sequence (CDS) of *CHR00029783,* named *CiCCoAOMT1,* was amplified using specific primers (Table S4), then inserted into the pET32a vector at the BamHI/XhoI sites. After sequencing confirmation, the *CiCCoAOMT1*-pET32a construct was transferred into *Escherichia coli* strain BL21 (DE3) for fusion protein expression. Transformants harboring *CiCCoAOMT1*-pET32a were cultivated until the OD_600_ reached 0.6 at 37 °C, and then induced with 0.5 mM isopropyl *β*-D-thiogalactoside (IPTG) at 16 °C for 24 h. The induced bacterial cells were harvested by centrifugation and then washed with cold 1× PBS buffer, resuspended, and sonicated on ice. Recombinant protein was purified by Ni-NTA gravity column (Sangon Biotech, Shanghai, China), and transferred to storage buffer (50 mM Tris-HCl, pH 8.0) using an Amicon-Ultra-0.5 Ultracel-10k membrane, and stored at −80 °C for further analysis.

### 4.10. Enzyme Assays and Analysis of C. indicum OMT Reaction Products

The enzymatic reactions (100 μL) included Tris–HCl buffer (50 mM, pH 8.0), 500 μM SAM, 200 μM substrate, and 25 μL of purified protein solution (2.5 mg/mL). The reactions were incubated at 37 °C for 2 h and quenched with the addition of double volume of methanol. The mixtures were filtered through 0.22 μm nylon columns and analyzed by HPLC.

### 4.11. Subcellular Localization

The coding regions of *CiCCoAOMT1* without the stop codon were constructed on the plant expression vector pCAMBIA1300-GFP. The recombinant construct (*CiCCoAOMT1*-GFP) and the empty vector (pCAMBIA1300-GFP) were transferred into *Agrobacterium tumefaciens* strains GV3101. The positive clones were cultured in liquid LB medium and then resuspended in MES buffer (10 mM MES, 10 mM MgCl_2_, 150 μM acetosyringone, pH 5.6) till OD_600_ reached 0.8. The suspension was infiltrated into *Nicotiana benthamiana* leaves after 2 h incubation. Images were captured by a Zeiss LSM800 confocal microscope 2 d after.

### 4.12. Overexpression of CiCCoAOMT1 in C. indicum In Vivo

*A*. *tumefaciens* strains EHA105 carrying the plasmid vector with *CiCCoAOMT1-GFP* genes were grown in LB (lysogeny broth) medium at 28 °C to OD_600_  =  0.6, and then centrifuged at 6000× *g* for 10 min and resuspended in an equal volume of MS (Murashige and Skoog) liquid medium as the infection solution. The empty vector pCAMBIA1300-cGFP was used as a control. The leaves of *C*. *indicum* tissue-culture seedlings cultured for about 35 days were used for infiltration, and inoculated on pre-culture medium (MS + 6-BA (6-Benzylaminopurine) 0.5 mg/L + NAA (1-Naphthylacetic acid) 1.0 mg/L) for 3 days in the dark. The leaf discs were immersed in the suspension for 10 min, and then placed on the co-cultured medium (MS + 6-BA 0.5 mg/L + NAA 1.0 mg/L + AS (Acetosyringone) 100 μM), and kept in the dark. After 2 days, the leaf discs were switched to be placed on the primary screening medium (MS + 6-BA 0.5 mg/L + NAA 1.0 mg/L + Carb 350 mg/L). When there was no *A*. *tumefaciens* outbreak after 7 days, the leaf discs were transferred to the screening medium 1 (MS + 6-BA 0.5 mg/L + NAA 1.0 mg/L + Carb 160 mg/L + Hyg 10 mg/L), and subcultured every 15 days. When no *A*. *tumefaciens* was observed, the leaf discs could be placed on the screening medium 2 (MS + 6-BA 0.5 mg/L + NAA 1.0 mg/L + Carb 80 mg/L + Hyg 8 mg/L). It lasted for about one month without *A*. *tumefaciens* outbreak. The screened calluses were collected, frozen in liquid nitrogen, and stored at −80 °C to analyze the gene expression and metabolites by qRT-PCR and HPLC-QTOF-MS.

### 4.13. Statistical Analyses

Statistical analysis was conducted using Microsoft Excel (version 2019). Data were obtained with three replicates. Figures exhibition was performed in GraphPad Prism (version 8).

## 5. Conclusions

In our study, a total of 79 *OMT*s were identified throughout the Chrysanthemum genome. The proteins encoded by these genes were distributed in two groups of the phylogenetic tree, which was supported by gene cluster analysis and conserved motifs distribution characteristics. RNA-Seq and qRT-PCR analysis suggested that these *OMT* genes exhibited tissue expression differences. Moreover, we found that *COMT*s were highly expressed in the capitulum, especially in full bloom, while *CCoAOMT*s were highly expressed in leaves. Additionally, we identified a true CCoAOMT enzyme, CiCCoAOMT1, and found that it catalyzes *O*-methylation of quercetin and luteolin at the 3′-OH position. The systematic exploration of the Chrysanthemum *OMT* gene family in this work provides clues to understanding the process of flavonoids *O*-methylation and regulation in *C*. *indicum*, as well as new ideas for screening *C*. *indicum* germplasm.

## Figures and Tables

**Table 1:** Discovered motifs in the amino sequences of *C*. *indicum* OMTs.

No. Motif	Logo	E-Value	Sites	Width
1	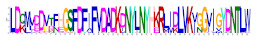	1.3 × 10^−1199^	30	50
2	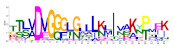	2.1 × 10^−744^	70	25
3	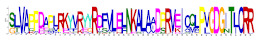	5.5 × 10^−851^	24	50
4	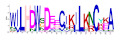	1.1 × 10^−550^	40	21
5	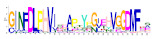	1.2 × 10^−585^	39	29
6	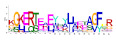	8.4 × 10^−545^	62	21
7	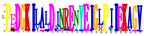	1.4 × 10^−544^	27	28
8	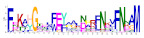	1.1 × 10^−498^	42	27
9	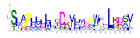	1.1 × 10^−307^	40	25
10	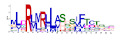	7.3 × 10^−297^	43	21
11	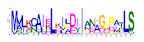	1.5 × 10^−310^	32	25
12	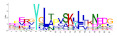	3.4 × 10^−273^	45	21
13	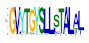	7.0 × 10^−261^	27	15
14	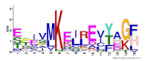	1.8 × 10^−226^	56	15

Sites, the number of sequences containing the motif; Width, the sequence length of the motif, measured in bp.

## Data Availability

No new data were created or analyzed in this study. Data sharing is not applicable to this article.

## Supplementary Materials

The following supporting information can be downloaded at: https://www.mdpi.com/article/10.3390/ijms251810037/s1.
