# Supplementary material for: Systematic Identification and Characterization of O-Methyltransferase Gene Family Members Involved in Flavonoid Biosynthesis in Chrysanthemum indicum L
Source: Int J Mol Sci. 2024 Sep 18;25(18):10037. doi: 10.3390/ijms251810037 (PMC11432614; doi:10.3390/ijms251810037)
Supplement: Supplementary file 1 [file ijms-25-10037-s001.zip › Supplementary Figure_IJMS.pdf]

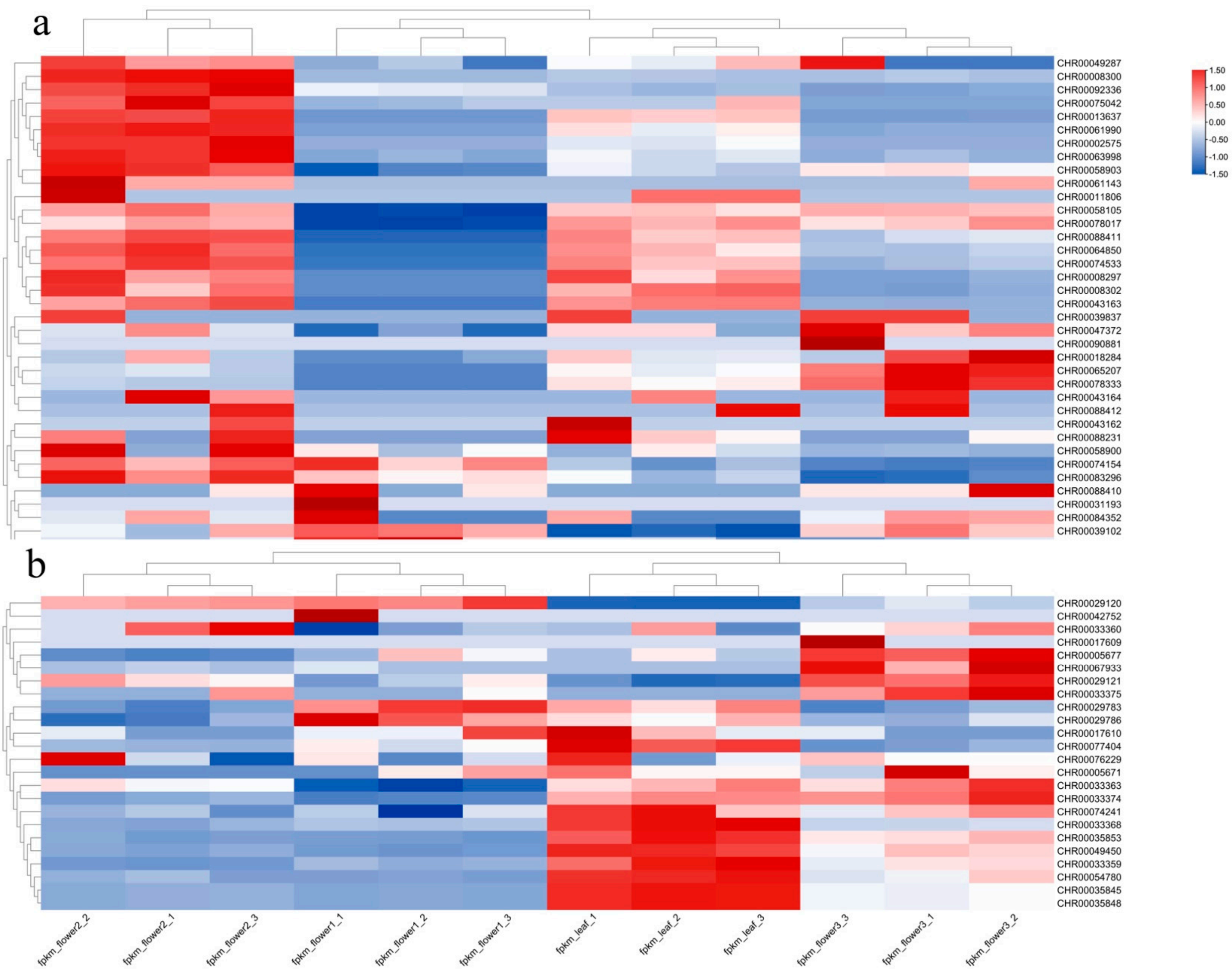


Figure S1. Agglomerative hierarchical clustering of *OMTs* in different capitulum development stages and leaves of *C. indicum*. a. *CiCOMT* genes; b. *CiCCoA* genes. The original data of the RNA-seq is shown in Tables S2. The color scale from blue to red color represents Z-score-normalized gene expression levels from low to high. The tree on the left and top of the heat map respectively shows hierarchical clustering between genes and hierarchical clustering between samples.

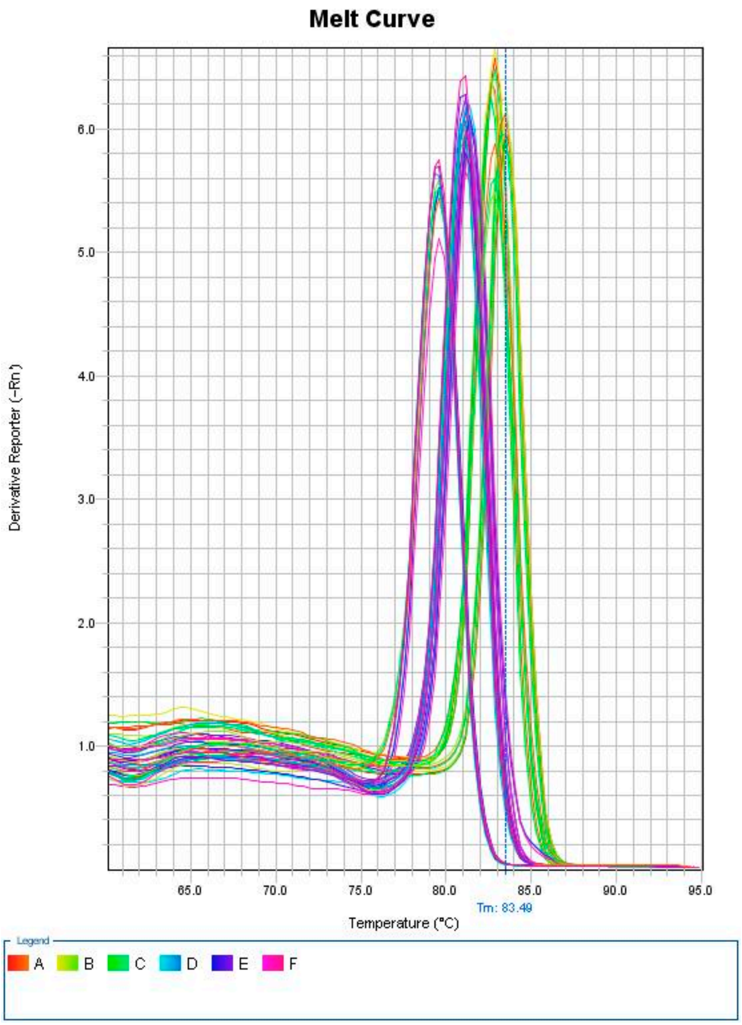


Figure S2. The qRT-PCR melting curve graph. The melting curve showed a single peak confirming the specificity of amplification.

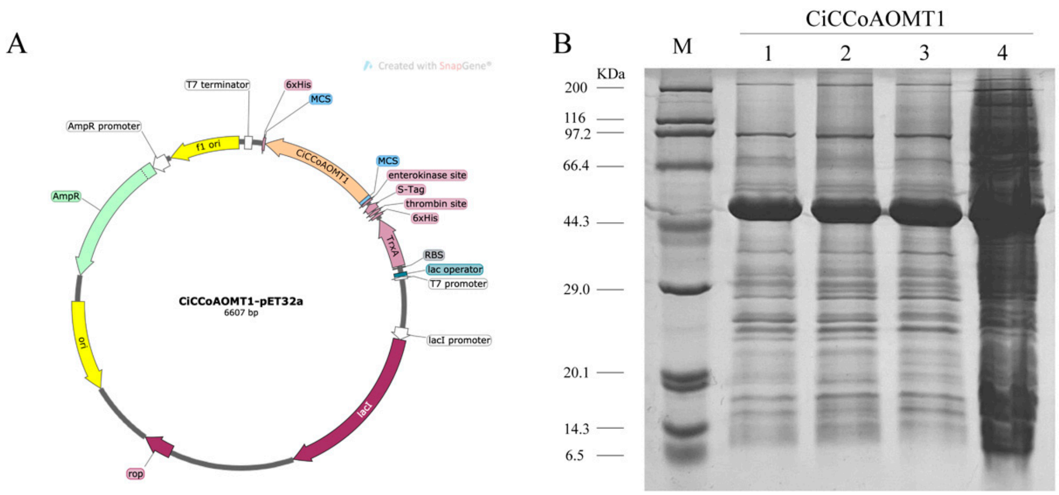


Figure S3. Expression of the *CiCCoAOMT1* gene in *E. coli*. (A) The full lengh ORF of *CiCCoAOMT1* were inserted into the pET32a vector under control of the T7 promoter. (B) SDS-PAGE analysis of expression and purification of CiCCoAOMT1-pET32a. Lane M: molecular weight marker; Lane 1-3: The purified protein; Lane 2: The crude protein induced by IPTG.
