# Supplementary material for: Systematic Identification and Characterization of O-Methyltransferase Gene Family Members Involved in Flavonoid Biosynthesis in Chrysanthemum indicum L
Source: Int J Mol Sci. 2024 Sep 18;25(18):10037. doi: 10.3390/ijms251810037 (PMC11432614; doi:10.3390/ijms251810037)
Supplement: Supplementary file 1 [file ijms-25-10037-s001.zip › Supplementary Table_IJMS.pdf]

Additional Table S1 List of all *COMT* and *CCoAOMT* genes identified in the Chrysanthemum genome

List of all *COMT* genes identified in the Chrysanthemum genome

Gene ID	Transcript length (bp)	Protein length (aa)	Exon numbers	Mw (kDa)	pI	Instability Index	GRAVY	Functional Domains (Start-End, aa)	Strand	Subcellular location
CHR00002575	1155	361	2	40.83	5.67	35.46	-0.096	139-342	+	cytoskeleton
CHR00008297	1197	372	2	41.92	6.1	35.18	-0.097	146-354	+	cytoplasm
CHR00008300	1197	371	2	41.68	6.01	34.57	-0.082	144-353	+	chloroplast
CHR00008302	1156	371	3	41.64	5.93	33.26	-0.072	144-353	+	cytoplasm
CHR00011806	1086	361	4	40.27	5.7	22.82	0.034	138-343	+	cytoplasm
CHR00013637	1170	363	2	40.85	6.01	33	-0.049	136-345	+	cytoskeleton
CHR00014258	714	237	2	26.42	5.32	36.39	-0.024	132-234	-	cytoplasm
CHR00018284	1103	355	4	39.68	5.44	25.69	-0.01	132-336	-	cytoplasm
CHR00031193	579	192	2	21.14	8.24	25.11	0.237	112-173	+	cytoplasm
CHR00039102	1159	367	4	40.62	5.44	34.01	-0.043	132-333	-	endoplasmic reticulum
CHR00039837	1002	333	2	37.14	5.21	39.67	0.05	106-314	-	cytoplasm
CHR00043161	1293	364	3	41.46	5.35	36.01	-0.136	135-346	-	cytoplasm
CHR00043162	1095	364	2	41.35	5.8	35.22	-0.045	169-346	-	cytoskeleton
CHR00043163	1101	366	2	41.65	5.59	32.62	-0.116	138-347	-	cytoskeleton
CHR00043164	1026	341	2	38.77	5.57	22.4	-0.111	113-322	-	nucleus
CHR00044867	1175	365	4	40.57	5.49	44.13	-0.052	137-344	-	chloroplast
CHR00044870	1197	365	4	40.78	5.3	43.74	-0.082	137-344	-	chloroplast
CHR00047372	1127	353	2	39.21	5.12	34.27	0.117	129-334	+	cytoplasm

CHR00049287	1120	328	2	36.13	6.36	37.76	0.023	129-296	+	cytoplasm
CHR00049601	879	292	3	32.52	4.77	38.91	-0.082	129-273	-	cytoplasm
CHR00053968	1134	346	2	38.99	5.45	28.96	-0.084	138-342	-	cytoskeleton
CHR00056557	930	309	3	35.27	5.57	**44.77**	-0.085	135-291	-	cytoplasm
CHR00058105	1110	348	4	38.71	5.81	28.69	-0.058	135-329	+	cytoskeleton
CHR00058900	1034	335	2	37.24	5.35	43.43	-0.008	129-316	-	cytoplasm
CHR00058903	1085	352	4	39.15	6.83	40.47	-0.174	163-333	-	cytoplasm
CHR00060717	1144	361	2	40.55	5.63	38.62	-0.102	137-343	-	cytoskeleton
CHR00061143	1123	370	5	41.55	5.22	37.71	-0.164	137-345	+	cytoskeleton
CHR00061990	814	259	2	28.52	4.67	34.57	0.164	142-244	+	chloroplast
CHR00063998	872	287	4	32.33	5.66	33.96	-0.021	64-269	+	cytoplasm
CHR00064850	1234	258	9	28.86	5.84	31.46	0.001	6-148	+	cytoplasm
CHR00065207	968	310	3	34.34	5.16	**22.31**	0.04	132-291	+	cytoplasm
CHR00071761	975	324	2	35.81	6.66	30.84	-0.074	138-265	-	cytoskeleton
CHR00071849	1155	384	2	43.21	6.46	32.65	-0.002	157-366	+	cytoplasm
CHR00074154	1095	364	3	39.64	5.07	26.91	0.01	141-345	+	chloroplast
CHR00074533	912	303	4	33.54	5.79	32.11	0.133	103-284	-	cytoskeleton
CHR00075042	1068	355	4	39.78	5.24	35.23	-0.014	132-337	-	cytoplasm
CHR00077883	1059	352	2	39.06	5.58	32.28	0.069	128-333	+	cytoplasm
CHR00078017	1117	350	4	38.79	5.74	27	0.013	127-331	+	cytoplasm
CHR00078333	1093	288	4	32.1	6.83	26.97	-0.075	65-269	+	chloroplast
CHR00083296	1448	351	5	39.31	5.82	43.6	-0.27	128-332	-	cytoplasm
CHR00084352	1062	353	2	39.31	5.67	37.19	-0.076	129-334	+	cytoplasm
CHR00088231	693	230	3	26.24	5.71	34	-0.164	8-137	+	cytoplasm
CHR00088410	1344	447	6	50	7.58	33.7	0.066	130-331	+	cytoplasm

CHR00088411	1294	350	4	38.99	5.51	32.3	0.09	127-332	+	cytoplasm
CHR00088412	609	202	4	22.44	5.15	24.51	0.046	13-202	+	cytoplasm
CHR00090881	934	287	2	32.37	5.67	34.31	-0.082	65-269	-	cytoplasm
CHR00092335	681	226	4	24.89	8.25	30.48	-0.121	4-207	+	cytoplasm
CHR00092336	1095	364	3	39.7	5.24	30.45	0.014	141-345	+	chloroplast

List of all *CCoAOMT* genes identified in the Chrysanthemum genome

**Gene ID**	**Transcript length (bp)**	**Protein length (aa)**	**Exon numbers**	**Mw (kDa)**	**pI**	**Instability Index**	**GRAVY**	**Functional Domains (Start-End, aa)**	**Strand**	**Subcellular location**
CHR00005671	808	235	5	26.52	5.45	28.83	-0.169	78-278	-	chloroplast
CHR00005677	704	229	5	25.88	5.05	29.33	-0.211	1-201	-	chloroplast
CHR00017609	693	230	5	26.2	5.53	32.11	-0.2	45-165	-	cytoskeleton
CHR00017610	723	240	5	27.15	5	31.76	-0.187	40-184	-	cytoskeleton
CHR00017611	636	211	4	23.96	4.5	25.32	-0.078	41-179	-	cytoskeleton
CHR00029120	708	235	5	26.36	5.47	26.62	-0.029	32-243	+	cytoskeleton
CHR00029121	747	202	5	23.11	4.83	34.81	-0.337	105-316	+	cytoskeleton
CHR00029783	822	247	4	27.87	5.2	36.09	-0.223	40-251	-	cytoskeleton
CHR00029786	744	247	4	27.66	5.28	31.36	-0.166	35-246	-	cytoskeleton
CHR00033359	806	244	5	27.4	5.17	31.55	-0.155	32-188	+	cytoplasm
CHR00033360	789	202	2	22.75	5.23	26.54	-0.037	32-189	+	cytoplasm
CHR00033361	561	186	2	21.36	8.48	25.64	-0.034	36-201	-	cytoplasm
CHR00033362	597	198	3	22.23	5	28.32	-0.173	32-196	+	cytoplasm
CHR00033363	677	200	2	22.37	4.83	32.56	-0.224	1-159	+	cytoplasm
CHR00033368	796	244	2	27.5	5.17	30.03	-0.202	32-207	+	cytoplasm

CHR00033374	826	244	2	27.46	5.27	31.44	-0.194	30-203	+	cytoplasm
CHR00033375	647	187	2	21.29	5.67	**24.98**	-0.081	116-302	+	cytoplasm
CHR00035843	968	208	2	23.5	5.79	33.94	-0.205	24-234	+	cytoplasm
CHR00035845	1031	244	3	27.39	5.29	34.85	-0.148	29-239	+	cytoplasm
CHR00035848	902	201	2	22.49	5.02	31.07	-0.187	24-234	+	cytoplasm
CHR00035851	815	243	2	27.26	5.71	29.62	-0.074	18-228	+	cytoplasm
CHR00035853	804	244	2	27.36	5.17	35.58	-0.156	18-229	+	cytoplasm
CHR00042752	558	185	3	20.88	6.3	40.65	0.078	32-188	+	cytoplasm
CHR00049450	954	317	2	35.43	5.79	36.05	-0.084	32-243	-	cytoplasm
CHR00054780	826	244	2	27.48	5.27	32.03	-0.172	32-243	+	cytoplasm
CHR00066976	1143	303	4	34.15	6.92	33.43	0.097	38-242	-	cytoplasm
CHR00067933	796	244	2	27.53	5.17	30.73	-0.203	32-243	-	cytoplasm
CHR00074241	1495	279	10	31.11	8.17	**44.28**	-0.073	32-243	-	cytoplasm
CHR00076229	570	189	2	21.12	4.81	32.49	-0.194	35-246	+	cytoplasm
CHR00077404	759	252	5	28.27	5.23	37.36	-0.234	32-243	-	endoplasmic reticulum
CHR00090571	528	175	3	20.24	9.88	41.61	-0.466	32-243	+	nucleus

Additional Table S2 Uniprot entries of proteins from other organisms used in phylogenetic analysis

Subfamily	Subgruop	Organism	UniProt entry	Protein name
CCoAOMT	PFOMTs	*Arabidopsis thaliana* (Mouse-ear cress)	Q9C5D7	AtCCoAMT
		Mesembryanthemum crystallinum (Common ice plant)	Q6YI95	McOMT
		Oryza sativa subsp. japonica (Rice)	Q9XGP7	OsROMT15
		Oryza sativa subsp. japonica (Rice)	Q7F8T6	OsROMT17
		Ocimum basilicum (Sweet basil)	S5DQS6	ObCCoAOMT
		Plagiochasma appendiculatum	A0A1P8NPM8	PaF6OMT
		Stellaria longipes (Longstalk starwort)	Q43161	SlCCoAOMT
		Vaccinium corymbosum (Highbush blueberry)	A0A385Z812	VcCCoAOMT
		Vanilla planifolia (Vanilla)	F2YP46	VpNOMT
	true CCoAOMTs	*Arabidopsis thaliana* (Mouse-ear cress)	O49499	AtCCoAOMT1

Carthamus tinctorius (Safflower)	B6F0U5	CtCoAOMT2
Carthamus tinctorius (Safflower)	B6F0U7	CtCoAOMT4
Carthamus tinctorius (Safflower)	B6F0U8	CtCoAOMT5
Carthamus tinctorius (Safflower)	B6F0U9	CtCoAOMT6
Eucalyptus globulus (Tasmanian blue gum)	O81185	EgCCOMT
Eucalyptus globulus (Tasmanian blue gum)	Q9SWB8	EgCCoAOMT2
Medicago sativa (Alfalfa)	Q40313	MsCCOMT
Mesembryanthemum crystallinum (Common ice plant)	O65162	McCCoAMT
Nicotiana tabacum (Common tobacco)	O04899	NtCCoAOMT5
Nicotiana tabacum (Common tobacco)	O24149	NtCCoAOMT2
Petroselinum crispum (Parsley)	P28034	PcCCoAOMT
Pinus taeda (Loblolly pine)	Q9ZTT5	PtCCoAOMT

	Plagiochasma appendiculatum	A0A0U2YR01	PaOMT1
	Plagiochasma appendiculatum	A0A173FEI7	PaOMT2
	Populus trichocarpa (Western balsam poplar)	O65862	PtCCoAOMT1
	Populus trichocarpa (Western balsam poplar)	O65922	PtCCoAOMT2
	Solanum tuberosum (Potato)	Q8H9B6	StCCoAOMT
	Vitis vinifera (Grape)	Q43237	VvCCoAMT
	Zea mays (Maize)	Q9XGD5	ZmCCoAOMT2
	Zinnia violacea (Garden zinnia)	Q41720	ZvCCoAOMT
	Arabidopsis thaliana (Mouse-ear cress)	O49499	AtCCoAOMT1
COMT	*Ammi majus* (Bishop's weed)	Q6T1F5	AmCOMT
	Arabidopsis thaliana (Mouse-ear cress)	Q9FK25	AtCOMT1
	Capsicum annuum (Capsicum pepper)	Q9FQY8	CaCOMT

Capsicum chinense (Scotch bonnet)	O81646	CcCOMT
Carthamus tinctorius (Safflower)	A0A0B6VJH3	CtAAOMT
Carthamus tinctorius (Safflower)	S6BHN8	CtMROMT
Carthamus tinctorius (Safflower)	S6AWZ4	CtFOMT
Catharanthus roseus (Madagascar periwinkle)	Q8W013	CrCOMT1
Chrysosplenium americanum (Golden saxifrage)	P59049	CaOMT1
Chrysosplenium americanum (Golden saxifrage)	Q42653	CaOMT2
Chrysosplenium americanum (Golden saxifrage)	Q42654	CaF3'OMT
Clarkia breweri (Fairy fans)	O23760	CbCOMT
Escovopsis weberi	A0A0M9VX70	EwFOMT
Eucalyptus globulus (Tasmanian blue gum)	Q9SWC2	EgCOMT1
Glycine max (Soybean)	C6TAY1	GmSOMT2

	Glycine soja (Wild soybean)	A0A0B2Q223	GsFOMT
	Medicago sativa (Alfalfa)	P28002	MsCOMT
	Mentha piperita (Peppermint)	Q6VMV8	MpOMT4
	Mentha piperita (Peppermint)	Q6VMW0	MpOMT2
	Ocimum basilicum (Sweet basil)	Q9XGV9	ObCOMT2
	Ocimum basilicum (Sweet basil)	Q9XGW0	ObCOMT1
	Ocimum basilicum (Sweet basil)	K0I210	ObFOMT4
	Ocimum basilicum (Sweet basil)	K0I977	ObFOMT1
	Ocimum basilicum (Sweet basil)	K0I986	ObFOMT6
	Ocimum basilicum (Sweet basil)	K0ICR0	ObFOMT5

Additional Table S3 FPKM values of *CiCOMT* and *CiCCoAOMT genes*

FPKM values of *CiCOMT* in different *Chrysanthemum indicum* tissues

Gene name	fpkm_flower1_1	fpkm_flower1_2	fpkm_flower1_3	fpkm_flower2_1	fpkm_flower2_2	fpkm_flower2_3	fpkm_flower3_1	fpkm_flower3_2	fpkm_flower3_3	fpkm_leaf_1	fpkm_leaf_2	fpkm_leaf_3	average_fpkm_flower1_	average_fpkm_flower2_	average_fpkm_flower3_	average_fpkm_leaf_
CHR00002575	0	0	0.08	10.4	9.53	13.35	41.36	51.4	41.59	0	0	0	0.026	11.093	44.783	0
CHR00008297	0.17	0.17	0.31	2.12	1.15	1.63	1.54	1.74	2.29	0	0	0	0.216	1.633	1.856	0
CHR00008300	5.15	2.41	2.38	4.41	4.13	2.37	74.87	77.73	69.88	0	0	0.07	3.313	3.636	74.16	0.023
CHR00008302	0.26	0.29	0.47	2.48	3.27	3.43	2.21	3.26	3.96	0	0	0	0.34	3.06	3.143	0
CHR00011806	0	0	0	0	0.09	0.09	0	0	0.18	0	0	0	0	0.06	0.06	0
CHR00013637	7	5.2	7.78	143.87	136.31	142.92	234.37	262.02	241.62	0	0	0	6.66	141.033	246.003	0
CHR00014258	0.15	0	0.3	0.29	0.27	0	0.15	0.31	0.29	1.34	0.97	0.48	0.15	0.186	0.25	0.93
CHR00018284	0.74	0.18	1.09	0.45	0.26	0.27	0.52	0.18	0.17	0	0	0.08	0.67	0.326	0.29	0.026
CHR00031193	0	0	0	0	0	0	0	0	0	0	0.18	0	0	0	0	0.06

CHR00039102	123.62	113.75	114.55	86.06	88.37	85.36	99.43	117.83	107.49	123.44	126.51	117.73	117.306	86.596	108.25	122.56
CHR00039837	0.1	0.1	0	0.1	0	0	0	0	0.1	0	0	0	0.066	0.033	0.033	0
CHR00043161	0.36	0.44	0.42	0.48	0.31	0.34	1.74	2.16	1	3.52	3.85	3.91	0.406	0.376	1.633	3.76
CHR00043162	0	0	0	0.17	0	0	0	0.09	0	0	0	0	0	0.056	0.03	0
CHR00043163	29.93	30.78	31.82	130.43	140.82	141.07	148.68	163.73	122.03	0	0	0	30.843	137.44	144.813	0
CHR00043164	0.16	0	0	0	0.11	0	0.19	0.1	0	0	0	0	0.053	0.036	0.096	0
CHR00044867	1.88	2.56	2.63	1.86	2.3	1.97	3.52	2.12	2.79	10.89	12.45	14.15	2.356	2.043	2.81	12.496
CHR00044870	5.42	5.59	6.05	5.1	5.04	5	5.44	4.21	5.24	7.67	8.48	9.01	5.686	5.046	4.963	8.386
CHR00047372	0.27	0.54	0.35	0.25	0.25	0.09	0.34	0.17	0.17	0.08	0	0	0.386	0.196	0.226	0.026
CHR00049287	0	0.62	0	0.26	0.23	0.37	0.42	0.44	0.56	0.16	0.1	0	0.206	0.286	0.473	0.086
CHR00049601	NA	NA	NA	NA	NA	NA	NA	NA	NA	NA	NA	NA	NA	NA	NA	NA
CHR00053968	0.26	0.18	0.09	0.08	0.33	0.09	0	0.17	0	0.62	0.8	0.95	0.176	0.166	0.056	0.79

CHR00056557	NA	NA	NA	NA	NA	NA	NA	NA	NA	NA	NA	NA	NA	NA	NA	NA
CHR00058105	8.21	8.29	7.8	7.59	7.78	6.97	9.98	8.44	8.58	0.43	0.18	0.13	8.1	7.446	9	0.246
CHR00058900	0.12	0.11	0.09	0.09	0.33	0.21	0.08	0.85	0.93	0.13	0.34	0.29	0.106	0.21	0.62	0.253
CHR00058903	6.27	6.06	5.35	5.21	4.47	3.96	10.23	9.25	10.8	2.01	0.9	2.09	5.893	4.546	10.093	1.666
CHR00060717	NA	NA	NA	NA	NA	NA	NA	NA	NA	NA	NA	NA	NA	NA	NA	NA
CHR00061143	0	0	0.09	0	0	0	0.09	0.09	0.25	0	0	0	0.03	0	0.143	0
CHR00061990	0.56	0.26	0.51	5.31	3.45	4.73	12.28	11.81	11.64	0	0	0	0.443	4.496	11.91	0
CHR00063998	0.6	0.12	0.23	1.96	1.25	1.56	6.04	7.1	6.43	0.34	0	0	0.316	1.59	6.523	0.113
CHR00064850	24.86	26.69	31.04	74.43	63.67	50.42	99.59	83.01	88.25	0.15	0.12	0.08	27.53	62.84	90.283	0.116
CHR00065207	3.87	2.6	3.4	1.47	1.18	1.38	1.12	0.82	0.97	0	0	0	3.29	1.343	0.97	0
CHR00071761	NA	NA	NA	NA	NA	NA	NA	NA	NA	NA	NA	NA	NA	NA	NA	NA
CHR00071849	NA	NA	NA	NA	NA	NA	NA	NA	NA	NA	NA	NA	NA	NA	NA	NA

CHR00074154	1.38	1.5	1.48	2.57	1.71	2.4	4.24	5.42	5.33	3.94	6	4.91	1.453	2.226	4.996	4.95
CHR00074533	88.84	75.23	106.55	296.39	227.47	233.23	372.89	341.91	310.52	0	0	0	90.206	252.363	341.773	0
CHR00075042	0	0	0	0.43	0.43	1.63	3.62	2.65	2.38	0.23	0.17	0.43	0	0.83	2.883	0.276
CHR00077883	0.19	0.19	0.19	0.09	0	0	0	0	0	0.76	0.43	0.43	0.19	0.03	0	0.54
CHR00078017	23.09	21.17	27.82	27.08	24.59	27.92	26.61	25.22	21.39	0.08	0.33	0.63	24.026	26.53	24.406	0.346
CHR00078333	15.82	11.4	13.5	6.98	5.82	6.54	3.34	3.3	3.83	0	0	0	13.573	6.446	3.49	0
CHR00083296	0.33	0.29	0.5	1.17	0.75	0.93	1.73	2.19	2.29	1.23	1.47	1.34	0.373	0.95	2.07	1.346
CHR00084352	0.19	0.1	0.18	0.18	0	0	0.18	0.09	0.09	0	0.34	0	0.156	0.06	0.12	0.113
CHR00088231	0	0	0.11	0.35	0.15	0.11	0	0.3	0.22	0	0	0	0.036	0.203	0.173	0
CHR00088410	0.07	0.07	0.21	0	0	0	0	0.07	0	0	0.2	0.07	0.116	0	0.023	0.09
CHR00088411	74.55	55.84	79.99	155.91	120.19	126.99	184.56	180.92	159.8	0	0.14	0.07	70.126	134.363	175.093	0.07
CHR00088412	0.19	0	0	0	0	0.19	0	0.18	0	0	0	0	0.063	0.063	0.06	0

CHR00090881	0	0.11	0	0	0	0	0	0	0	0	0	0	0.036	0	0	0
CHR00092335	NA	NA	NA	NA	NA	NA	NA	NA	NA	NA	NA	NA	NA	NA	NA	NA
CHR00092336	2.46	2.38	3.43	5.43	4.47	5.2	26.47	31.93	24.36	9.22	9.89	8.65	2.756	5.033	27.586	9.253

FPKM values of *CiCCoAOMT* in different *Chrysanthemum indicum* tissues

Gene name	fpkm_flower1_1	fpkm_flower1_2	fpkm_flower1_3	fpkm_flower2_1	fpkm_flower2_2	fpkm_flower2_3	fpkm_flower3_1	fpkm_flower3_2	fpkm_flower3_3	fpkm_leaf_1	fpkm_leaf_2	fpkm_leaf_3	average_fpkm_flower1	average_fpkm_flower2	average_fpkm_flower3	average_fpkm_leaf
CHR00005671	0	0.26	0.38	0	0	0	0.77	0.25	0.13	0.46	0.24	0.24	0.213	0	0.383	0.313
CHR00005677	2.33	3.61	3.02	1.77	1.88	1.85	4.42	5.21	4.72	2.45	3.23	2.53	2.986	1.833	4.783	2.736
CHR00017609	0	0	0	0	0	0	0	0	0.15	0	0	0	0	0	0.05	0
CHR00017610	0.15	0.15	0.44	0	0.14	0	0	0	0.14	0.66	0.27	0.14	0.246	0.046	0.046	0.356
CHR00017611	NA	NA	NA	NA	NA	NA	NA	NA	NA	NA	NA	NA	NA	NA	NA	NA
CHR00029120	23.57	22.58	28	20.79	19.57	21.56	11.91	9.61	9.38	0.14	0	0	24.716	20.64	10.3	0.046
CHR00029121	1.24	1.88	2.67	2.83	3.5	2.58	3.94	4.77	4.28	1.16	0.64	0.68	1.93	2.97	4.33	0.826

CHR00029783	74.73	87.74	89.6	32.31	37.56	39.7	38.62	43.51	32.98	70.75	62.57	76.67	84.023	36.523	38.37	69.996
CHR00029786	9.12	7.47	6.48	3.17	2.98	4.16	4.01	4.87	4.14	5.73	5.25	6.29	7.69	3.436	4.34	5.756
CHR00033359	18.27	14.97	16.39	10.06	10.79	10.94	37.95	39.5	29.24	58.37	72.62	77.21	16.543	10.596	35.563	69.4
CHR00033360	2.67	4.09	4.75	7.53	5.1	8.65	6.1	7.13	5.6	4.61	6.79	3.77	3.836	7.093	6.276	5.056
CHR00033361	NA	NA	NA	NA	NA	NA	NA	NA	NA	NA	NA	NA	NA	NA	NA	NA
CHR00033362	NA	NA	NA	NA	NA	NA	NA	NA	NA	NA	NA	NA	NA	NA	NA	NA
CHR00033363	9.55	8.05	9.92	19.07	20.99	19.3	25.69	29.49	20.94	21.07	22.94	25.77	9.173	19.786	25.373	23.26
CHR00033368	5.91	5.39	6.23	1.61	2.12	3.82	8.3	9.22	7.94	33.89	38.08	40.52	5.843	2.516	8.486	37.496
CHR00033374	0.28	0.77	1.06	3.59	2.48	4.42	18.22	22.86	16.39	14.62	17.14	16.9	0.703	3.496	19.156	16.22
CHR00033375	0	0	0.17	0	0	0.34	0.49	0.66	0.33	0	0	0	0.056	0.113	0.493	0
CHR00035843	NA	NA	NA	NA	NA	NA	NA	NA	NA	NA	NA	NA	NA	NA	NA	NA
CHR00035845	0.28	0.63	0.58	1.45	0.9	1.68	8.89	10.82	9.54	29.98	31.77	31.58	0.496	1.343	9.75	31.11

CHR00035848	0.28	0.63	0.58	1.45	0.9	1.68	8.89	10.82	9.54	29.98	31.77	31.58	0.496	1.343	9.75	31.11
CHR00035851	NA	NA	NA	NA	NA	NA	NA	NA	NA	NA	NA	NA	NA	NA	NA	NA
CHR00035853	2	1.83	1.29	1.7	2.96	2.15	12.66	15.54	12.02	21.83	26.75	24.61	1.706	2.27	13.406	24.396
CHR00042752	0.21	0	0	0	0	0	0	0	0	0	0	0	0.07	0	0	0
CHR00049450	1.8	1.33	1.76	2.39	2.83	3.15	13.16	11.78	8.79	22.34	21.98	20.43	1.63	2.79	11.243	21.583
CHR00054780	0.74	0.9	0.69	1.94	1.38	0.72	4.09	6.21	3.42	10.96	11.25	11.88	0.776	1.346	4.573	11.363
CHR00066976	NA	NA	NA	NA	NA	NA	NA	NA	NA	NA	NA	NA	NA	NA	NA	NA
CHR00067933	0.35	0	0	0.16	0	0	1.13	2.95	2.27	0	0	0	0.116	0.053	2.116	0
CHR00074241	1.61	1.04	1.7	1.59	1.51	1.37	1.98	2.17	1.72	2.4	2.52	1.98	1.45	1.49	1.956	2.3
CHR00076229	2.64	0.66	1.9	1.88	5.64	0	2.4	2.38	3.04	4.86	0.94	2.2	1.733	2.506	2.606	2.666
CHR00077404	66.26	55.42	63.63	45.81	47.16	45.72	41.17	46.43	38.47	117.01	94.26	99.6	61.77	46.23	42.023	103.623
CHR00090571	NA	NA	NA	NA	NA	NA	NA	NA	NA	NA	NA	NA	NA	NA	NA	NA

Additional Table S4 List of primers used in this study

**Primer Name**	**Primer Sequence (5'-3')**	**Primer Used For**
GAPDH-qF	GGGGATTGATTTGGTGATAG	qRT-PCR analysis
GAPDH-qR	AATGTCTTCGGAATGGTTGT	
EF-1α-qF	GGTCAGATTGGAAACGGTTAT	
EF-2α-qR	AGGTGGGTATTCAGCAAAGG	
CHR00029120-qF	AGATATGGACCTGGAGGCGTATGAG	
CHR00029120-qR	AGCCGGTAGAGCCTGAGATTCG	
CHR00043163-qF	TGTTGCGGACATGAAGGATACTACG	
CHR00043163-qR	TCAACACTTCTTCGTCTCCCCAATC	
CHR00044867-qF	AGCATGTTGGAGGAGATATGTTCGC	
CHR00044867-qR	CCACCACCACCTTACCGCATTC	
CHR00058903-qF	AGTCCATCAACCAGATTGCG	
CHR00058903-qR	ACCATCCTTGCTAATGGTGC	
CHR00064850-qF	GCAGCAATAGATGCACGGTTCAATG	
CHR00064850-qR	AGACCACCTCCAACATCTACTAGCC	
CHR00074533-qF	GCAGCAATAGATGCACGGTTCAATG	
CHR00074533-qR	AGACCGCCTCCAACATCTACTAGC	
CHR00078017-qF	GGTCGATGTAGGAGGTGGTCTAGG	
CHR00078017-qR	CAGGATACAGTGGTGCATGACGAG	
CHR00088411-qF	GATGCCCGCTTCAATGACGTTTTC	
CHR00088411-qR	ACCGCCTCCAACATCTACTAGCC	
CiCCoAOMT1-F	ATGGCAACAGTCAACGAAAGCAG	cDNA amplification
CiCCoAOMT1-R	TTAGCTTATGCGGCGACATAGAGT	
CiCCoAOMT1-EGFP-F	acacgggggacgagctc ATGGCAACAGTCAACGAAAGCAG	Subcellular localization
CiCCoAOMT1-EGFP-R	gcccttgctcaccatgtcgacGCTTATGCGGCGACATAGAGT	

Additional Table S5 Concentrations of flavonoids in the *Chrysanthemum indicum* tissues

Compounds	flower1-1 (mg/g)	flower1-2 (mg/g)	flower1-3 (mg/g)	flower2-1 (mg/g)	flower2-2 (mg/g)	flower2-3 (mg/g)	flower3-1 (mg/g)	flower3-2 (mg/g)	flower3-3 (mg/g)
Isorhamnetin	8.39E-01	7.90E-01	8.36E-01	8.29E-01	8.91E-01	8.20E-01	4.91E-01	3.43E-01	4.00E-01
Luteolin	2.09E+00	2.20E+00	2.17E+00	1.66E+00	1.76E+00	1.62E+00	1.03E+00	7.02E-01	8.12E-01
Quercitrin	8.91E-02	1.20E-01	1.28E-01	6.69E-02	6.44E-02	6.38E-02	7.56E-02	6.31E-02	5.53E-02
Linarin	2.97E+01	2.86E+01	2.96E+01	2.52E+01	2.69E+01	2.49E+01	2.61E+01	1.85E+01	2.12E+01
Luteolin	5.56E-01	2.30E-01	2.20E-01	2.90E-01	3.18E-01	2.70E-01	4.02E-01	2.62E-01	3.02E-01
Quercetin	5.81E-02	7.45E-01	7.57E-01	5.45E-01	6.26E-01	4.94E-01	5.14E-01	3.16E-01	3.45E-01
Apigenin	2.25E-01	1.77E-01	1.87E-01	1.70E-01	1.73E-01	1.54E-01	3.49E-01	2.24E-01	2.72E-01
Naringenin	9.30E-02	1.49E-01	1.56E-01	2.32E-01	2.40E-01	1.98E-01	2.11E-01	1.43E-01	1.47E-01
Kaempferol	1.91E-01	1.81E-01	1.86E-01	2.38E-01	2.49E-01	2.12E-01	3.35E-01	2.52E-01	2.93E-01
Acacetin	7.42E-02	7.22E-02	6.60E-02	6.68E-02	7.10E-02	6.37E-02	1.20E-01	8.58E-02	9.75E-02
Total flavonoids	1.53E+02	1.48E+02	1.44E+02	1.21E+02	1.28E+02	1.29E+02	1.02E+02	1.08E+02	1.04E+02
